# Accuracy of the Instantaneous Breathing and Heart Rates Estimated by Smartphone Inertial Units

**DOI:** 10.3390/s25041094

**Published:** 2025-02-12

**Authors:** Eliana Cinotti, Jessica Centracchio, Salvatore Parlato, Daniele Esposito, Antonio Fratini, Paolo Bifulco, Emilio Andreozzi

**Affiliations:** 1 Department of Electrical Engineering and Information Technologies, University of Naples Federico II, Via Claudio, 21, I-80125 Naples, Italy; eliana.cinotti@unina.it (E.C.); salvatore.parlato@unina.it (S.P.); emilio.andreozzi@unina.it (E.A.); 2 Department of Information and Electrical Engineering and Applied Mathematics, University of Salerno, Via Giovanni Paolo II, 132, I-84084 Fisciano, Italy; daesposito@unisa.it; 3 College of Engineering and Physical Sciences, Aston University, Birmingham B4 7ET, UK; a.fratini@aston.ac.uk

**Keywords:** gyrocardiography, seismocardiography, heartbeat detection, respiration, heart rate, breathing rate, smartphone, accelerometer, gyroscope, cardiorespiratory monitoring

## Abstract

Seismocardiography (SCG) and Gyrocardiography (GCG) use lightweight, miniaturized accelerometers and gyroscopes to record, respectively, cardiac-induced linear accelerations and angular velocities of the chest wall. These inertial sensors are also sensitive to thoracic movements with respiration, which cause baseline wanderings in SCG and GCG signals. Nowadays, accelerometers and gyroscopes are widely integrated into smartphones, thus increasing the potential of SCG and GCG as cardiorespiratory monitoring tools. This study investigates the accuracy of smartphone inertial sensors in simultaneously measuring instantaneous heart rates and breathing rates. Smartphone-derived SCG and GCG signals were acquired from 10 healthy subjects at rest. The performances of heartbeats and respiratory acts detection, as well as of inter-beat intervals (IBIs) and inter-breath intervals (IBrIs) estimation, were evaluated for both SCG and GCG via the comparison with simultaneous electrocardiography and respiration belt signals. Heartbeats were detected with a sensitivity and positive predictive value (PPV) of 89.3% and 93.3% in SCG signals and of 97.3% and 97.9% in GCG signals. Moreover, IBIs measurements reported strong linear relationships (R^2^ > 0.999), non-significant biases, and Bland–Altman limits of agreement (LoA) of ±7.33 ms for SCG and ±5.22 ms for GCG. On the other hand, respiratory acts detection scored a sensitivity and PPV of 95.6% and 94.7% for SCG and of 95.7% and 92.0% for GCG. Furthermore, high R^2^ values (0.976 and 0.968, respectively), non-significant biases, and an LoA of ±0.558 s for SCG and ±0.749 s for GCG were achieved for IBrIs estimates. The results of this study confirm that smartphone inertial sensors can provide accurate measurements of both instantaneous heart rate and breathing rate without the need for additional devices.

## 1. Introduction

Cardiac monitoring is one of the commonly used analyses in a comprehensive picture of an individual’s health and its evolution, and it is generally performed via the analysis of electrocardiography (ECG) signals. However, ECG signals do not carry information on the mechanical functioning of the heart, for which ultrasounds are used as an elective modality. Previous research demonstrated that it is possible to extract heart-related mechanical features examining other types of signals, such as seismocardiograms and gyrocardiograms [1,2,3,4,5,6,7,8,9,10,11,12,13,14]. Seismocardiography (SCG) and Gyrocardiography (GCG) are non-invasive techniques that record, unlike ECG-based electrical monitoring, small vibrations of the chest wall generated by heart contractions via inertial sensors [4,5,6,15,16,17]. SCG and GCG use lightweight, miniaturized, three-axis accelerometers and gyroscopes, respectively, to monitor cardiac-induced linear accelerations and angular velocities of the precordium [12,13,18,19]. Since they are applied onto subjects’ chests, these sensors are also capable of monitoring thoracic movements induced by respiration [20,21,22,23,24,25,26]. For this reason, relevant information about both cardiac and respiratory activity, primarily one’s heart rate and breathing rate, can be extracted from accelerometric and gyroscopic signals [27,28,29,30].

Monitoring the heart and breathing rates is a crucial aspect of a more complete view of a patient’s health, and the literature is plenty on the topic. By analyzing the heartbeat, cardiac disorders, such as heart failure and arrhythmias, can be recognised. Moreover, changes in the breathing rate can indicate both respiratory and cardiac issues, such as heart failure or asthma. Among the most common tests based on the observation of respiration is sleep analysis, which is useful for detecting, for example, sleep apnoea [31,32], so monitoring must be constant in order to diagnose and treat potential disorders. Heartbeats are usually detected on SCG and GCG signals by locating specific peaks and valleys, which mainly correspond to the opening and closure of heart valves, while respiration is obtained by capturing baseline wanderings in accelerometric and gyroscopic signals [4,5,6,7,12].

Cardiorespiratory diseases and their consequences still affect a considerable percentage of the population; therefore, health monitoring can benefit by seamlessly extracting information from commonly used personal devices towards a quicker recognition of acute conditions or developing pathologies. This allows the general population to become more informed, better manage their physical conditions, and maintain more autonomy. Smartphones have become a daily companion alongside daily life activities, the most used personal devices this far, and it is possible to exploit electronic components already present within these devices to evaluate subjects’ physical conditions outside clinical settings [33,34]. SCG- and GCG-based monitoring can be performed using accelerometers and gyroscopes integrated into any smartphone. To acquire these signals, the smartphone trivially needs to be placed on a subject’s chest. The use of a smartphone as a monitoring device allows subjects not to carry additional hardware, thus being more convenient and cost-effective; indeed, there is no need to purchase further devices. This approach also benefits doctors, who can remotely monitor patients’ cardiorespiratory activity in real time by taking advantage of data transmission over the network. In the case of anomalies, the doctor can act more quickly by having a complete overview of patients’ health conditions. The possibility to perform intermittent measurements during daily life is crucial for patients because, in the case of cardiovascular disorders, such as atrial fibrillation, a timely intervention is necessary since the duration of an arrhythmic event is short and difficult to record. Indeed, consulting a specialist and undergoing periodic measurements do not guarantee that atrial fibrillation episodes will occur during the check-up [35,36,37].

The use of smartphone inertial sensors has been investigated in recent years for the acquisition of cardiomechanical signals, particularly SCG and GCG [1,33,34,38]. Various studies have been presented to monitor vital signs, mainly the heart rate and breathing rate, from smartphone-derived SCG and GCG signals [18,19,39]. Of these, some studies focused only on heart rate estimation [2,34,40]; other studies only addressed breathing rate estimation [41]. Moreover, a comparison between accelerometers and gyroscopes in terms of the estimation accuracy of both the heart rate and breathing rate has never been carried out. Furthermore, some studies provided only measurements of the mean heart rate [42,43] or mean breathing rate [44]. In addition, many studies are lacking in accurate performance analysis with respect to reference techniques [41,45,46]. Finally, some studies proposed rather complex approaches to detecting heartbeats or respiratory acts in SCG or GCG signals [2,17,44].

To the best of our knowledge, the use of smartphone inertial sensors to simultaneously measure the instantaneous heart rate and breathing rate from both accelerometric and gyroscopic signals has never been proposed. This study addressed this issue. To this end, experimental tests were carried out on a cohort of 10 healthy subjects at rest in a supine position under quiet breathing. Smartphone-derived accelerometric and gyroscopic signals were recorded using an open-source app. The performances of heartbeats and respiratory acts detection, as well as of inter-beat intervals (IBIs) and inter-breath intervals (IBrIs) estimation, were evaluated against reference techniques via statistical analyses. Specifically, ECG signals were assumed as the reference for cardiac activity, while respiration belt signals were the ground truth for respiratory activity.

## 2. Materials and Methods

### 2.1. Signals Acquisition and Pre-Processing

**Figure 1:**
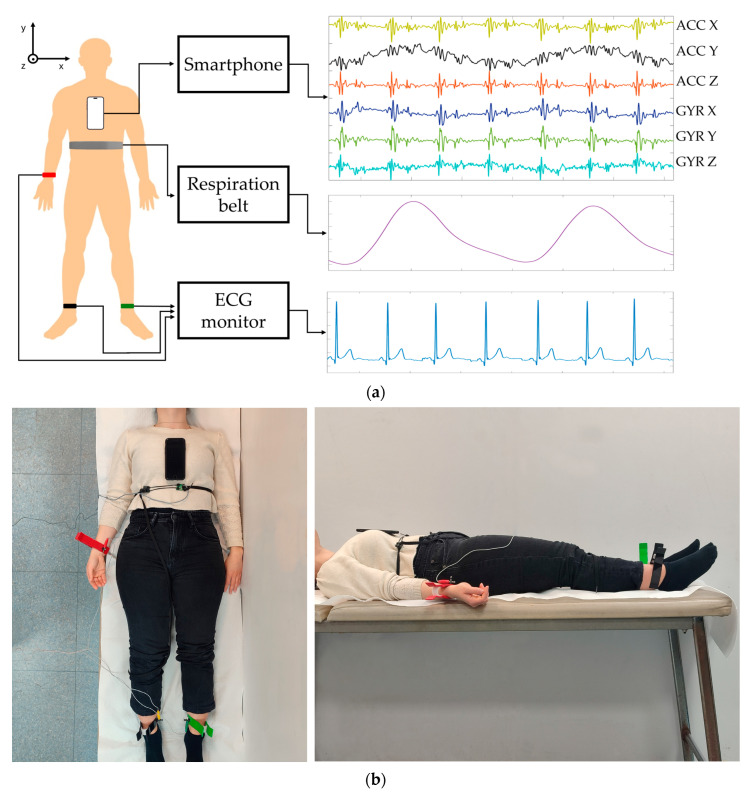
(**a**) Measurement setup: the subject lies supine on a bed in horizontal position while breathing at a natural pace. The smartphone is placed onto the subject’s chest along the sternum. The ERB is secured around the upper abdomen of the subject. ECG electrodes are positioned on the subject to record the ECG lead II via an ECG monitor. Triaxial ACC and GYR signals are acquired by the smartphone and, simultaneously, ECG and ERB signals are acquired via a separate data acquisition board. Examples of acquired signals are also depicted. (**b**) Two photos: on the left, a top view, and on the right, a side view of an actual subject equipped with the measurement setup.

Ten healthy subjects (eight males and two females, aged 25.75 ± 4.96 years) were involved in this study, who signed informed consent. The participants were asked to breathe at their natural pace while lying supine on a bed in a horizontal position. SCG and GCG signals were recorded using an iPhone 8 smartphone (Apple, Los Altos, CA, USA) placed on the subjects’ chest along the midline. An open-source app, namely, Phyphox (RWTH Aachen University) [47], was used to acquire three-axis accelerometric (ACC) and gyroscopic (GYR) signals from the smartphone inertial measurement unit at a sampling frequency of 100 Hz. Simultaneously, ECG lead II and respiration signals were acquired using a WelchAllyn Propaq^®^ Encore monitor and an electro-resistive band (ERB) [48], respectively, to provide reference measurements. The ECG and ERB signals were recorded via a National Instrument NI-USB6212 DAQ board (National Instruments Corp., Austin, TX, USA) at a sampling frequency of 10 kHz and 16-bit precision. Synchronization between the signals acquired via the smartphone (ACC and GYR) and the signals acquired via the acquisition board (ECG and ERB) was obtained by causing a brisk motion artifact. The motion artifact was generated by giving a small and sudden push to the subject’s body. Thus, both ACC and ECG signals were strongly corrupted by a short and large transient. Then, the first heartbeat after the transient was localized in both ACC and ECG signals, thus allowing for a beat-wise alignment of the two signal groups. This procedure allowed the same starting heartbeat to be recognized in the two signal groups. This ensured that all the following cardiac inter-beat and respiratory inter-breath intervals were aligned and suitable for comparison via the statistical analyses. Figure 1 shows the measurement setup adopted in the experimental tests. Specifically, the dorso-ventral *z*-axis ACC signals and the cranio-caudal *y*-axis GYR signals were considered for cardiac activity, while the cranio-caudal *y*-axis ACC and GYR signals were considered for respiratory activity. These signals were linearly interpolated at 10 kHz via the Matlab^®^ function “*interp1*” to obtain the same temporal resolution of ECG and ERB signals. Moreover, the ECG signal was first band-pass filtered in the 0.5–40 Hz frequency range via a fourth-order zero-lag Butterworth filter; then, a notch comb filter was used to remove the 50 Hz powerline interference and its higher harmonics. In this study, Matlab^®^ R2022a (The MathWorks, Inc., 1 Apple Hill Drive, Natick, MA, USA) was used for all processing operations.

### 2.2. Cardiac Activity Analysis

**Figure 2:**
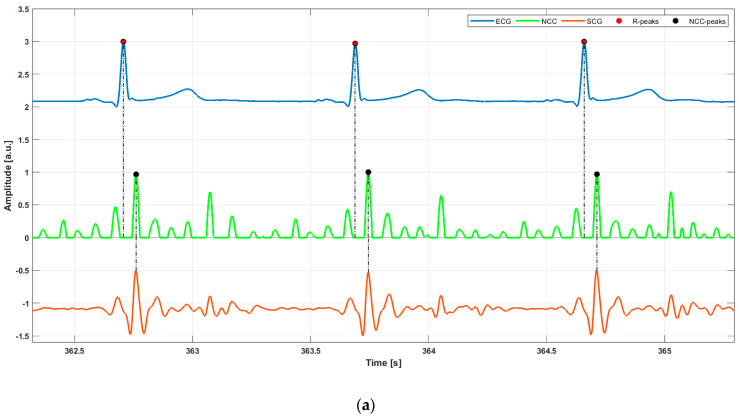

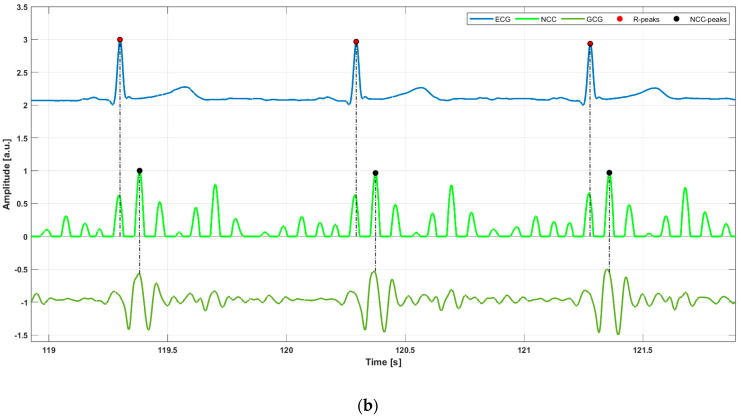
Some excerpts of ECG (blue line), NCC (light green line), and (**a**) SCG signal (orange line), (**b**) GCG signal (green line) from subject #5. Red and black points mark the locations of R-peaks and NCC-peaks, respectively.

**Figure 3:**
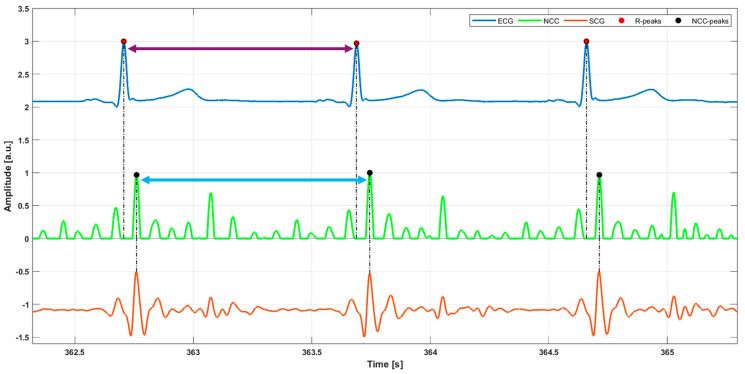
An example of IBI estimation in ECG and SCG signals of subject #5. For the ECG signal, the IBI is estimated as the temporal distance between two consecutive R-peaks (violet double arrow), while the same IBI is estimated for the NCC signal as the temporal distance between the two corresponding successive NCC-peaks (blue double arrow).

The *z*-axis SCG and *y*-axis GCG signals were obtained by band-pass filtering, respectively, the raw *z*-axis ACC and *y*-axis GYR signals via a second-order zero-lag Butterworth band-pass filter with cut-off frequencies of 7 and 30 Hz. To localize heartbeats on SCG and GCG signals, an ECG-free template matching method, widely presented in previous studies [49,50,51,52,53,54,55], was used. This technique involves selecting a heartbeat template from the signal to be analyzed and calculating the normalized cross-correlation (NCC) function between the selected template and the whole signal as a similarity measure. In this study, the template was manually selected from SCG and GCG signals, including both systolic and diastolic cardiac complexes, as in [49,50,51,52,53,54,55]. Specifically, the template was selected from two to three oscillations before the systolic peak (local maximum), where the amplitude of the oscillations was significantly reduced compared with the amplitude of the systolic peak and ended just after the last oscillation of the diastolic complex. High NCC values indicate high similarity between the selected template and the signal chunks; therefore, the NCC local maxima were considered as heartbeats’ fiducial points. On the other hand, to obtain reference heartbeats, R-peaks were located on the ECG signal using the well-known Pan–Tompkins algorithm [56], which is implemented in RunBioSigKit Matlab^®^ toolbox [57]. Figure 2 shows an example of heartbeats’ localization on ECG, SCG, and GCG signals from subject #5. True positives (TPs), false positives (FPs,) and false negatives (FNs) were annotated for both SCG and GCG signals with respect to reference R-peaks. In detail, heartbeats detected on SCG/GCG signals that corresponded to R-peaks on ECG signals were considered as TPs, heartbeats identified on SCG/GCG signals that did not correspond to R-peaks on ECG signals were marked as FPs, and missed heartbeats on SCG/GCG signals at locations where R-peaks were identified on ECG signals were counted as FNs. Finally, IBIs were calculated for the three signals as time differences between their consecutive heartbeat fiducial points (see Figure 3).

### 2.3. Respiratory Activity Analysis

**Figure 4:**
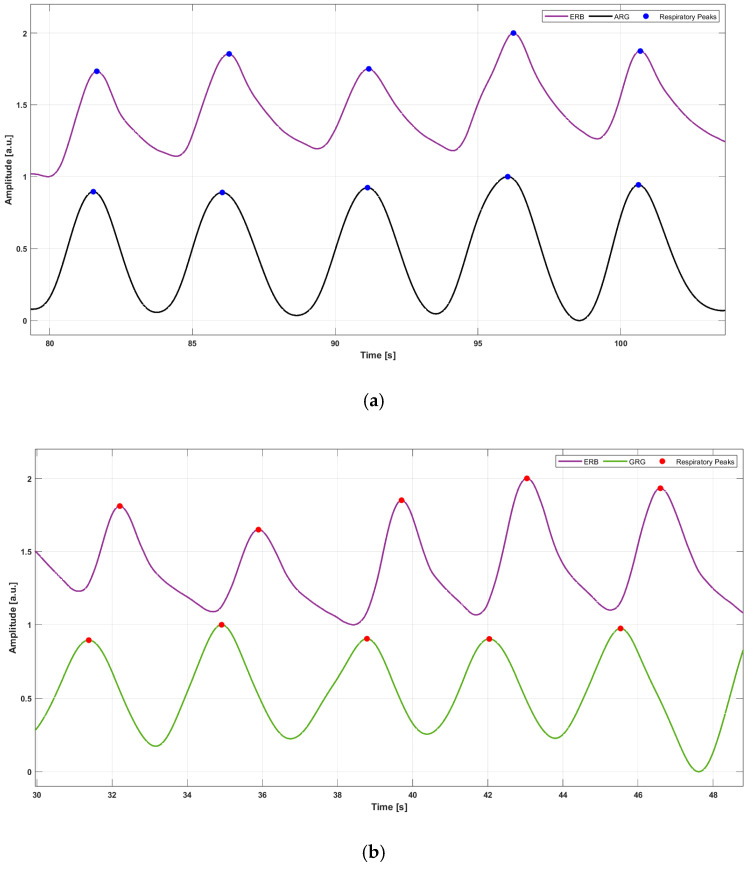
Some excerpts of ERB (purple line) signal and (**a**) ARG signal (black line), (**b**) GRG signal (green line) from subject #5. Blue points mark the locations of respiratory peaks on ERB/ARG signals, while red points mark the locations of respiratory peaks on ERB/GRG signals.

**Figure 5:**
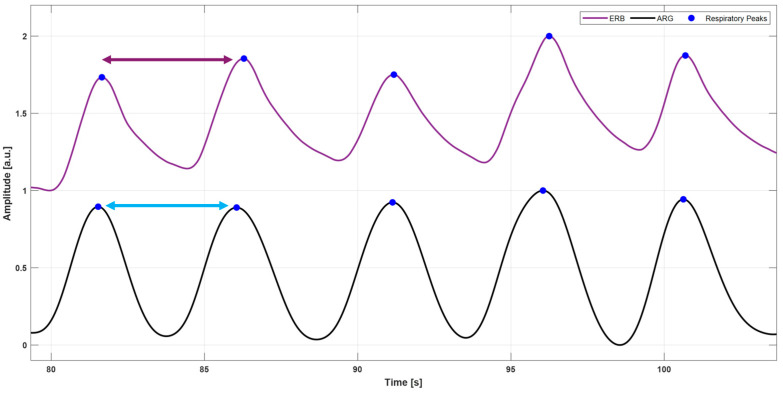
An example of IBrI estimation in ERB and ARG signals of subject #5. For the ERB signal, the IBrI is estimated as the temporal distance between two consecutive respiratory peaks (violet double arrow), while the same IBrI is estimated for the ARG signal as the temporal distance between the two corresponding successive respiratory peaks (blue double arrow).

Purely respiration signals, referred to as accelerometer-derived respiration (ARG) and gyroscope-derived respiration (GRG), were obtained, respectively, from the raw *y*-axis ACC and *y*-axis GYR signals by capturing their baseline wanderings due to respiratory-induced thoracic movements. These movements cause changes in the inclination and angular velocity of the smartphone accelerometer and gyroscope, respectively. These large respiratory components were extracted by applying third-order Savitzki–Golay filters [58], with frame lengths ranging from 1.5 to 10 s. This procedure helps to maintain the true shape of the ARG and GRG signals, unlike simple low-pass filtering, which may eliminate important higher-frequency components, particularly during forceful inspirations or expirations, and introduce artifacts into the resulting signals. The same processing was applied to the ERB signal, which was filtered via a third-order Savitzky–Golay filter with a frame length corresponding to about a 1.5 s interval. Then, respiratory acts were located on the three signals via the MATLAB^®^ function “*findpeaks*” by appropriately setting the minimum peak prominence and distance. Figure 4 shows an example of respiratory acts detection on the ERB signals and ARG and GRG signals. A comparison with respect to reference respiratory acts was performed for both ARG and GRG signals to annotate TPs, FPs, and FNs. In particular, respiratory peaks on ARG/GRG signals that matched references peaks on ERB signals were marked as TPs, respiratory acts on ARG/GRG signals that did not match reference peaks on ERB signals were annotated as FPs, and missed respiratory peaks on ARG/GRG signals with respect to reference peaks on ERB signals were counted as FNs. Finally, IBrIs measurements were obtained from the three signals by computing the time differences between consecutive respiratory acts, as depicted in Figure 5.

### 2.4. Statistical Analyses

To assess the accuracy of heartbeats and respiratory acts detections with respect to reference ECG and ERB signals, the sensitivity and positive predictive value (PPV) were considered as performance evaluation metrics and computed according to the following equations:

(1) $$Sensitivity\;(\%)=\frac{TP}{TP+FN}\;100$$

(2) $$PPV\;(\%)=\frac{TP}{TP+FP}\;100$$

where TP, FP, and FN indicate the number of true positives, false positives, and false negatives, respectively. Linear regression, correlation, and Bland–Altman analyses [59] were carried out to compare IBIs obtained from SCG and GCG signals with those provided by reference ECG and IBrIs obtained from ARG and GRG signals with those provided by reference ERB signals. These analyses were performed by using the MATLAB^®^ function “bland-altman-and-correlation-plot” [60]. IBIs and IBrIs corrupted by FPs and FNs were excluded from these analyses.

## 3. Results

### 3.1. Performance of Heartbeats’ Localization

**Figure 6:**
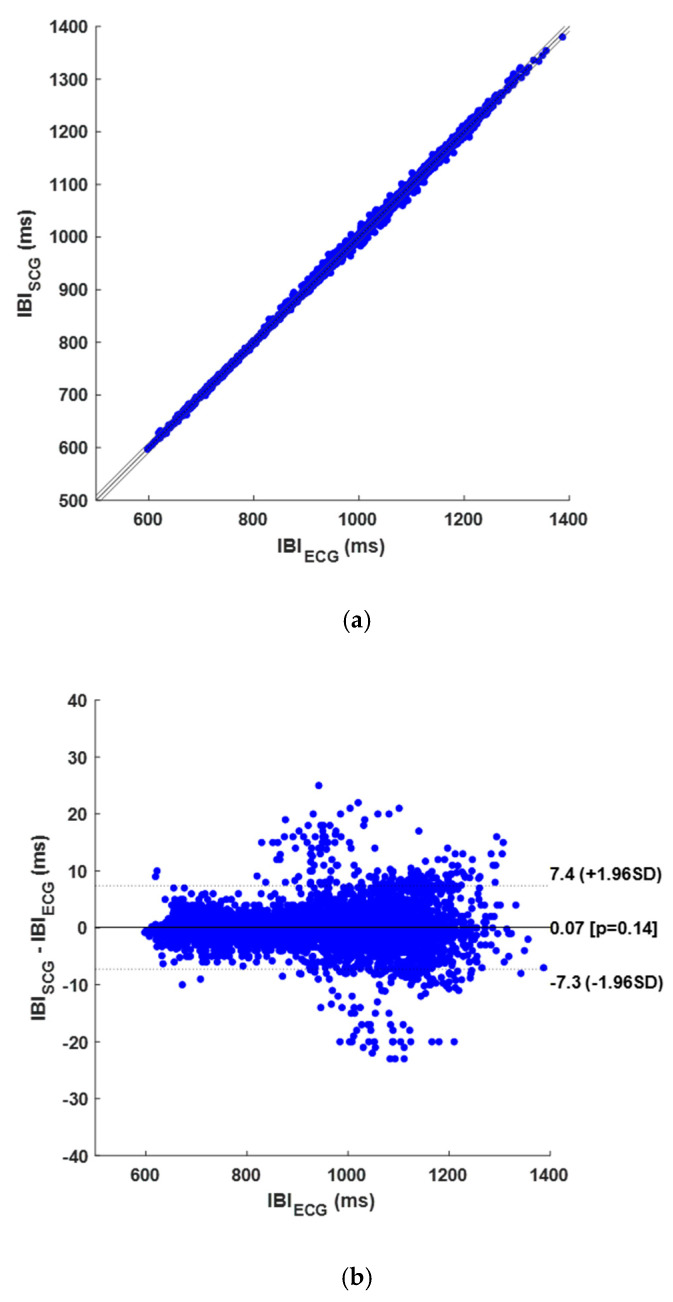
Results of statistical analyses on IBIs obtained from SCG signals: (**a**) correlation and linear regression plot; (**b**) Bland–Altman plot.

Table A1 in Appendix A reports the number of TPs, FPs, and FNs detected on SCG signals for each subject, along with the number of reference R-peaks. This table also indicates the number of compared IBIs. Across all subjects, 5660 heartbeats were identified in the reference ECG signals, while 5048 TPs, 360 FPs, and 608 FNs were recognized in the SCG signals. Therefore, the SCG signals scored a sensitivity of 89.3% and a PPV of 93.3%. Moreover, 5370 IBIs were compared via the correlation, linear regression, and Bland–Altman analysis. The regression and correlation analysis yielded a unitary slope and an intercept of 0.041 ms, with a coefficient of determination R^2^ > 0.999, as shown in Figure 6a. The Bland–Altman analysis revealed an LoA of ±7.33 ms and a non-significant bias (*p*-value of 0.142), as shown in Figure 6b. The results of the statistical analyses are also summarized in Table 1.

**Figure 7:**
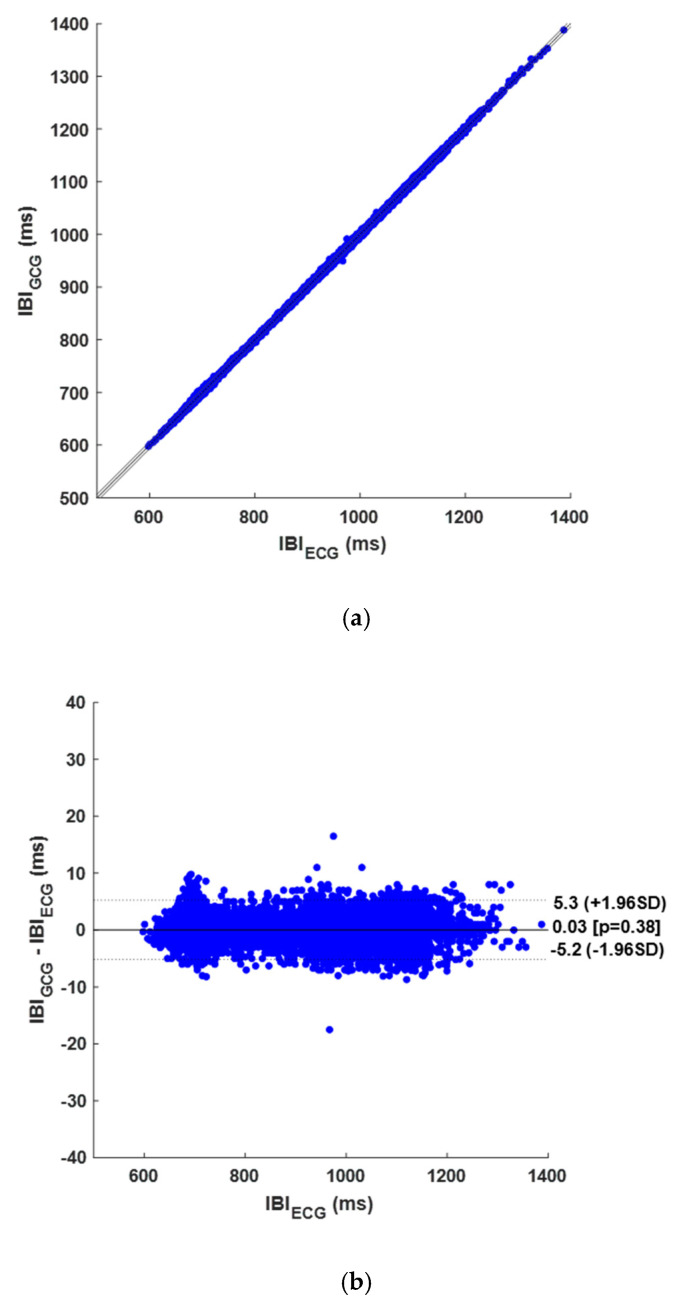
Results of statistical analyses on IBIs obtained from GCG signals: (**a**) correlation and linear regression plot; (**b**) Bland–Altman plot.

On the other hand, a total of 5506 TPs, 119 FPs, and 154 FNs were identified in the GCG signals, and 5370 IBIs were considered for correlation, linear regression, and Bland–Altman analysis. The results of the heartbeats detection per subject are shown in Table A2 in Appendix A. The GCG signals achieved a sensitivity of 97.3% and a PPV of 97.9% in heartbeats detection. Furthermore, the regression and correlation analysis on IBIs resulted in a unitary slope and an intercept of −0.648 ms (R^2^ > 0.999), as depicted in Figure 7a. The Bland–Altman analysis reported an LoA of ±5.22 ms and a non-significant bias (*p*-value of 0.378), as illustrated in Figure 7b. A summary of these results is also provided in Table 1.

### 3.2. Performance of Respiratory Acts Localization

**Figure 8:**
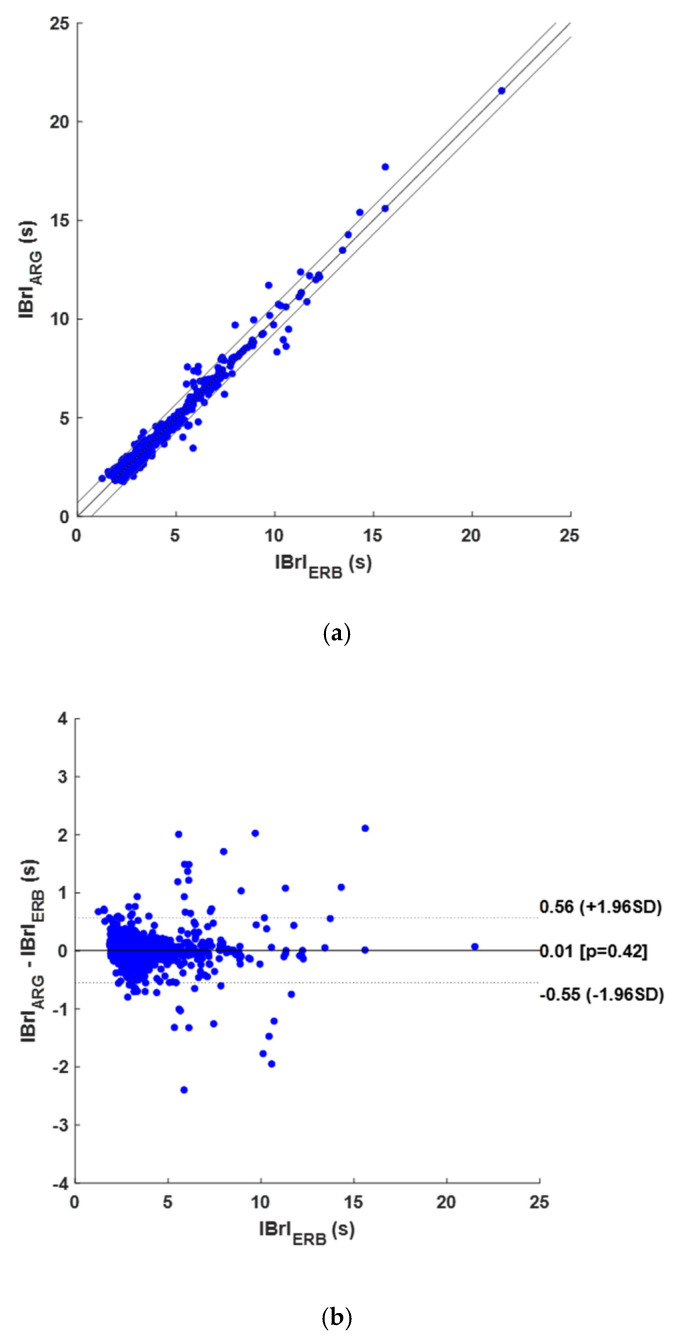
Results of statistical analyses on IBrIs obtained from ARG signals: (**a**) correlation and linear regression plot; (**b**) Bland–Altman plot.

Across all subjects, a total of 1315 respiratory acts were identified in the reference ERB signals, while 1190 TPs, 67 FPs, and 55 FNs were detected on the ARG signals reported, which scored a sensitivity of 95.6% and a PPV of 94.7%. The results of the respiratory acts detection per subject are indicated in Table A3 in Appendix A. Moreover, 1198 IBrI measurements were statistically compared. In detail, a unitary slope and an intercept of 0.0017 s were found in the regression and correlation analysis, with an R^2^ value of 0.976, as shown in Figure 8a. The Bland–Altman analysis reported an LoA of ±0.558 s and a non-significant bias (*p*-value of 0.423), as depicted in Figure 8b.

**Figure 9:**
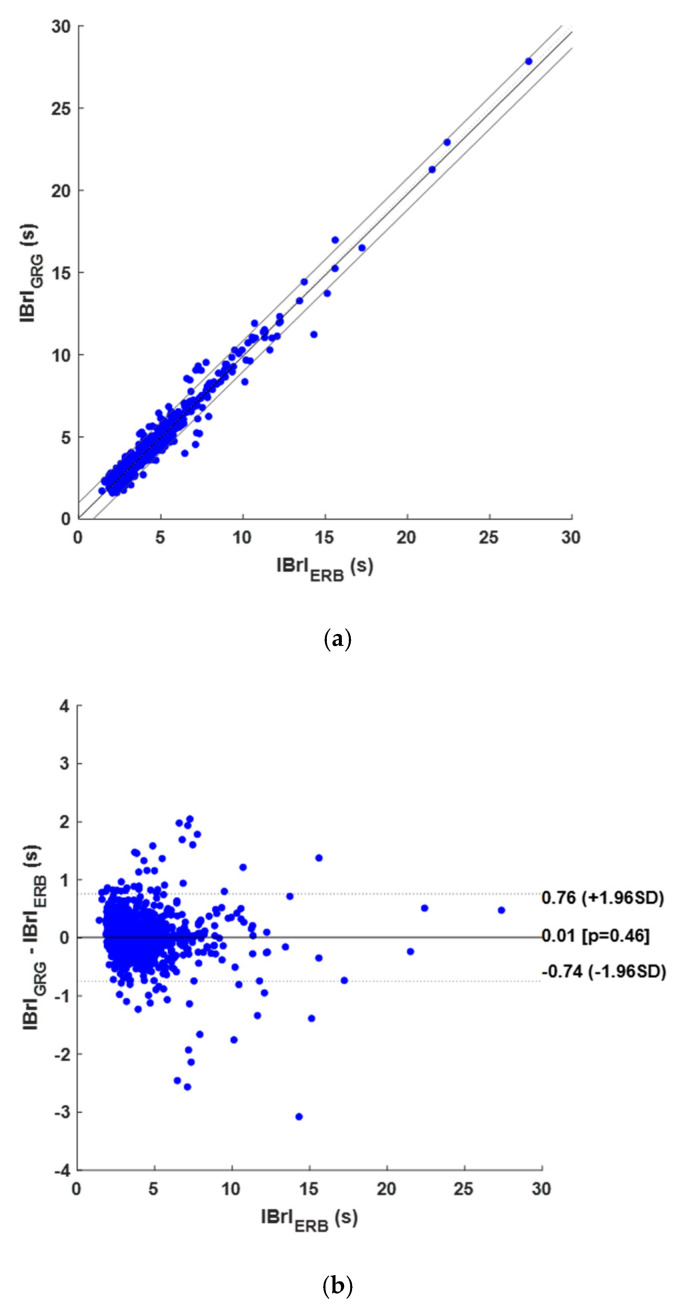
Results of statistical analyses on IBrIs obtained from GRG signals: (**a**) correlation and linear regression plot; (**b**) Bland–Altman plot.

On the other hand, 1154 TPs, 100 FPs, and 52 FNs were detected in the GRG signals, thus achieving a sensitivity of 95.7% and a PPV of 92.0%. The details are shown in Table A4 in Appendix A. The correlation and linear regression analysis, performed on 1211 IBrIs, reported a slope of 0.986 and an intercept of 0.065 s, with an R^2^ value of 0.968, as shown in Figure 9a, while an LoA of ±0.775 s and a non-significant bias (*p*-value of 0.461) resulted from the Bland–Altman analysis, as illustrated in Figure 9b. A summary of these statistical findings is provided in Table 2.

## 4. Discussion

This study demonstrated that instantaneous heart and breathing rates can be accurately estimated from accelerometric and gyroscopic signals acquired via inertial sensors embedded in a smartphone. Specifically, heartbeats were identified on these signals via an ECG-free template matching method, well-documented in previous studies [49,50,51,52,53,54,55], while respiratory acts were monitored by tracking thoracic movements, which reflect on accelerometric and gyroscopic signals as baseline wanderings. The results of the correlation and regression analyses showed a high degree of correlation between the inter-beat intervals estimated from smartphone-derived SCG and GCG signals and those obtained from simultaneous ECG signals (R^2^ > 0.999 for both SCG and GCG). Moreover, the Bland–Altman analyses reported non-significant biases and limits of agreement of ±7.33 ms for SCG and ±5.22 ms for GCG, thus suggesting that accurate measurements of instantaneous heart rate can be obtained from both SCG and GCG signals recorded via a smartphone, without the support of a concurrent ECG tracing. Similarly, the results of correlation and regression analyses performed on the inter-breath intervals scored R^2^ values of 0.976 for ARG and 0.968 for GRG with respect to those provided by simultaneous respiration signals, while the Bland–Altman analyses reported non-significant biases and limits of agreement of ±0.558 s for ARG and ±0.749 s for GRG, indicating that smartphone-derived accelerometric and gyroscopic signals also provide high accuracy in the estimation of instantaneous breathing rates.

These findings highlight that a smartphone can be used as a personal monitoring device. Indeed, it offers a user-friendly, cost-effective, and comfortable solution for obtaining relevant information about cardiorespiratory activity and accurately monitoring two vital signs, i.e., heart rate and breathing rate, without the need for additional, more expensive and obtrusive instruments. No other studies in the literature addressed the simultaneous estimation of instantaneous heart and breathing rates from both smartphone-derived SCG and GCG signals. Indeed, some studies focused only on mean heart rate or breathing rate estimation [1,33,36,40,44,61], and other studies measured only instantaneous heart rate or breathing rate alone, also obtaining much larger limits of agreement [2,34,41,42,43], while further studies did not even compare with reference techniques or provide the results of the performance analysis [40,41,46,62].

This study has some limitations. Data were collected from a small cohort of healthy subjects. The performance of the proposed monitoring method should be assessed on a larger cohort of subjects, possibly also including pathological subjects with cardiac and respiratory impairments. In addition, measurements were obtained only from subjects at rest, in supine position during quiet breathing. Securing the smartphone to the subjects’ chest, e.g., via an elastic band, could improve the robustness regarding motion artifacts and also allow for its use in various activities of daily living, involving different body postures and movements. Moreover, all data acquisitions were only performed via an iPhone8 smartphone. Further tests should be carried out on various smartphone models to ensure that accurate results could be obtained with any smartphone. This could enable the potential use of the proposed smartphone-based approach by a large number of smartphone users. As an example, this could be used in emergency situations by paramedics to obtain information about subjects’ health conditions in a timely manner. Another limitation is that the template matching approach requires the manual selection of the heartbeat template, which depends on the expertise of the operator. A promising future direction is the development of a fully automated template matching algorithm, which could also be integrated into a dedicated smartphone app in order to provide measurements of instantaneous heart rate and breathing rate in real time.

## Figures and Tables

**Table 1:** Results of statistical analyses performed on IBIs obtained from SCG and GCG signals.

		SCG	GCG
**Sample size**	**Subjects**	10	10
	**Compared IBI**	4710	5370
**Performance of heartbeats detection**	**Sensitivity (%)**	89.3	97.3
	**PPV (%)**	93.3	97.9
**Results of regression analysis**	**Slope**	1.00	1.00
	**Intercept (ms)**	0.041	−0.648
	**R^2^**	0.9996	0.9998
**Results of correlation analysis**	**r**	0.9998	0.9999
**Results of Bland–Altman analysis**	**Bias (ms)**	0.075	0.032
	**LoA(ms)**	7.33	5.22

**Table 2:** Results of statistical analyses performed on IBrIs obtained from ARG and GRG signals.

		ARG	GRG
**Sample size**	**Subjects**	10	10
	**Compared IBrI**	1198	1211
**Performance of respiratory acts detection**	**Sensitivity (%)**	95.6	95.7
	**PPV (%)**	94.7	92.0
**Results of regression analysis**	**Slope**	1.00	0.986
	**Intercept (s)**	0.002	0.065
	**R^2^**	0.976	0.968
**Results of correlation analysis**	**r**	0.988	0.984
**Results of Bland–Altman analysis**	**Bias (s)**	0.007	0.008
	**LoA(s)**	0.558	0.749

## Data Availability

The data presented in this article are not publicly available. Requests to access the data should be directed to the corresponding author.

## Appendix A

**Table A1:** Number of R-peaks detected in ECG signals, TPs, FPs, and FNs identified in SCG signals, along with the number of compared IBIs.

Subject ID#	R-Peaks	TP	FP	FN	Compared IBI
1	863	810	16	51	775
2	861	860	0	1	857
3	649	578	68	71	517
4	403	400	3	3	396
5	646	646	0	0	645
6	420	359	148	61	310
7	331	309	3	21	289
8	496	492	0	4	486
9	401	396	0	5	390
10	590	198	122	391	45
Total	5560	5048	360	608	4710

**Table A2:** Number of R-peaks detected in ECG signals and TPs, FPs, and FNs identified in GCG signals, along with the number of compared IBIs.

Subject ID#	R-Peaks	TP	FP	FN	Compared IBI
1	863	863	0	0	862
2	861	859	0	2	855
3	649	514	83	135	408
4	403	396	8	7	391
5	646	646	0	0	645
6	420	417	5	3	412
7	331	331	14	0	329
8	496	489	4	7	481
9	401	401	1	0	399
10	590	590	4	0	588
Total	5660	5506	119	154	5370

**Table A3:** Number of respiratory peaks detected in ERB signals and TPs, FPs, and FNs identified in ARG signals, along with the number of compared IBrIs.

Subject ID#	Reference Respiratory Acts	TP	FP	FN	Compared IBrI
1	196	195	0	1	193
2	116	98	15	3	111
3	238	232	3	4	230
4	40	23	5	6	21
5	158	158	0	0	157
6	104	94	5	5	94
7	56	40	9	7	43
8	153	116	16	25	108
9	125	122	3	0	124
10	129	112	11	4	117
Total	1315	1190	67	55	1198

**Table A4:** Number of respiratory peaks detected in ERB signals and TPs, FPs, and FNs identified in GRG signals, along with the number of compared IBrIs.

Subject ID#	Reference Respiratory Acts	TP	FP	FN	Compared IBrI
1	196	187	5	4	188
2	116	94	19	3	109
3	238	230	4	4	234
4	40	22	13	2	34
5	158	151	7	0	157
6	104	92	6	5	98
7	56	54	1	1	53
8	153	115	16	20	113
9	125	110	8	7	113
10	129	99	21	6	112
Total	1315	1154	100	52	1211

## Appendix B

In this study, several acronyms are used. Table A5 presents a complete list of the acronyms used.

**Table A5:** Complete list of acronyms and their meanings.

Acronym	Full Form
**ACC**	Accelerometer
**ARG**	Accelerometer-derived Respiration
**ECG**	Electrocardiography
**ERB**	Electro-resistive Respiratory Band
**FN**	False Negative
**FP**	False Positive
**GCG**	Gyrocardiography
**GRG**	Gyroscope-derived Respiration
**GYR**	Gyroscope
**IBI**	Inter-beat interval
**IBrI**	Inter-breath interval
**LoA**	Limits of Agreement
**NCC**	Normalized Cross-Correlation
**PPV**	Positive Predictive Value
**SCG**	Seismocardiography
**TN**	True Negative
**TP**	True Positive
